# Supplementary material for: A New Strategy for the Old Challenge of Thalidomide: Systems Biology Prioritization of Potential Immunomodulatory Drug (IMiD)-Targeted Transcription Factors
Source: Int J Mol Sci. 2023 Jul 15;24(14):11515. doi: 10.3390/ijms241411515 (PMC10380514; doi:10.3390/ijms241411515)
Supplement: Supplementary file 1 [file ijms-24-11515-s001.zip › Kowalski et al., 2023 - Supplemental Material 1.pdf]

PubMed:

Drug

Thalidomide[Mh] OR Thalidomide[Tiab] OR Sedoval[Tiab] OR Thalomid[Tiab] OR pomalidomide[Tiab] OR Lenalidomide[Mh] OR Lenalidomide[Tiab] OR Revlimid[Tiab] OR Revimid[Tiab]

AND

Molecular alteration (gene ou protein)

Transcriptome[Tiab] OR Transcriptome*[Tiab] OR Transcriptome Profile*[Tiab] OR Microarray Analysis[Mh] OR Microarray Analysis[Tiab] OR Epigenome[Mh] OR Epigenome*[Tiab] OR DNA Methylome*[Tiab] OR Methylome*[Tiab] OR Epigenomics[Mh] OR Epigenomic*[Tiab] OR Proteome[Mh] OR Proteome*[Tiab] OR Gene Expression[Mh] OR Gene Expression*[Tiab] OR Gene Expression Profiling[Mh] OR Gene Expression Profiling*[Tiab] OR Developmental Gene Expression Regulation*[Tiab] OR Embryologic Gene Expression Regulation*[Tiab] OR Gene Expression Regulation[Mh] OR Gene Expression Regulation*[Tiab] OR Transcriptome Profiling*[Tiab] OR Polymerase Chain Reaction[Mh] OR Polymerase Chain Reaction[Tiab] OR PCR[Tiab] OR Nested PCR[Tiab] OR Real-Time Polymerase Chain Reaction[Mh] OR Real-Time Polymerase Chain Reaction[Tiab] OR Real-Time PCR[Tiab] OR Real Time PCR[Tiab] OR Quantitative Real-Time Polymerase Chain Reaction[Tiab] OR Quantitative Real Time Polymerase Chain Reaction[Tiab] OR Quantitative Real-Time PCR[Tiab] OR Protein expression*[Tiab] OR Quantitative assay*[Tiab] OR In Situ Hybridization[Mh] OR In Situ Hybridization[Tiab] OR Hybridization in Situ[Tiab]

AND

Embryonic model

Stem Cells[Mh] OR Stem Cells*[Tiab] OR ProgenitOR Cells[Tiab] OR Mother Cells[Tiab] OR Induced Pluripotent Stem Cells[Mh] OR Induced Pluripotent Stem Cell*[Tiab] OR IPS Cells*[Tiab] OR Human Induced Pluripotent Stem Cell*[Tiab] OR hiPSC*[Tiab] OR Fibroblast-Derived Induced Pluripotent Stem Cell*[Tiab] OR Fibroblast-Derived IPS Cell*[Tiab] OR Fibroblast Derived IPS Cells*[Tiab] OR Fetal Stem Cells[Mh] OR Fetal Stem Cell*[Tiab] OR Hematopoietic Stem Cells[Mh] OR Hematopoietic Stem Cell*[Tiab] OR Hematopoietic ProgenitOR Cell*[Tiab] OR Lymphoid ProgenitOR Cells[Mh] OR Lymphoid ProgenitOR Cell*[Tiab] OR Lymphoid Stem Cell*[Tiab] OR Myeloid ProgenitOR Cells[Mh] OR Myeloid ProgenitOR Cell*[Tiab] OR Myeloid Stem Cell*[Tiab] OR Neural Stem Cells[Mh] OR Neural Stem Cell*[Tiab] OR Multipotent Stem Cells[Mh] OR Multipotent Stem Cell*[Tiab] OR Mesenchymal Stem Cells[Mh] OR Mesenchymal Stem Cell*[Tiab] OR Bone Marrow Mesenchymal Stem Cell*[Tiab] OR Bone Marrow Stromal Cell*[Tiab] OR Multipotent Bone Marrow Stromal Cell*[Tiab] OR Adipose-Derived Mesenchymal Stem Cell*[Tiab] OR Adipose Derived Mesenchymal Stem Cell*[Tiab] OR Adipose Tissue-Derived Mesenchymal Stem Cell*[Tiab] OR Adipose Tissue Derived Mesenchymal Stem Cell*[Tiab] OR Mesenchymal Stromal Cell*[Tiab] OR Multipotent Mesenchymal Stromal Cell*[Tiab] OR Mesenchymal ProgenitOR Cell*[Tiab] OR Wharton Jelly Cell*[Tiab] OR Wharton's Jelly Cell*[Tiab] OR Pluripotent Stem Cells[Mh] OR Pluripotent Stem Cell*[Tiab] OR Embryonic Stem Cells[Mh] OR Embryonic Stem Cell*[Tiab] OR Human Embryonic Stem Cells[Mh] OR Human Embryonic Stem Cell*[Tiab] OR hESC*[Tiab] OR H1-hESC*[Tiab] OR H9-hESC*[Tiab] OR Totipotent Stem Cells[Mh] OR Totipotent Stem Cell*[Tiab] OR Human Umbilical Vein Endothelial Cells[Mh] OR Human Umbilical Vein Endothelial Cell*[Tiab] OR HUVEC Cell*[Tiab] OR Embryoid Bodies[Mh] OR Embryoid Bod*[Tiab]

EMBASE:

Drug

‘Thalidomide’/exp OR ‘Sedoval’/exp OR ‘Thalomid’/exp OR ‘pomalidomide’/exp OR ‘Lenalidomide’/exp OR ‘Revlimid’/exp OR ‘Revimid’/exp

AND

Molecular alteration (gene ou protein)

‘Transcriptome’/exp OR ‘Transcriptomes’/exp OR ‘Transcriptome Profile’/exp OR ‘Transcriptome Profiles’/exp OR ‘Microarray Analysis’/exp OR ‘Microarray Analyzes’/exp OR ‘Epigenome’/exp OR ‘Epigenomes’/exp OR ‘DNA Methylome’/exp OR ‘DNA Methylomes’/exp OR ‘Methylome’/exp OR ‘Methylomes’/exp OR ‘Epigenomics’/exp OR ‘Epigenomic’/exp OR ‘Proteome’/exp OR ‘Proteomes’/exp OR ‘Gene Expression’/exp OR ‘Gene Expression Profiling’/exp OR ‘Developmental Gene Expression Regulation’/exp OR ‘Embryologic Gene Expression Regulation’/exp OR ‘Gene Expression Regulation’/exp OR ‘Transcriptome Profiling’/exp OR ‘Polymerase Chain Reaction’/exp OR ‘PCR’/exp OR ‘Nested PCR’/exp OR ‘Real-Time Polymerase Chain Reaction’/exp OR ‘Real-Time PCR’/exp OR ‘Real Time PCR’/exp OR ‘Quantitative Real-Time Polymerase Chain Reaction’/exp OR ‘Quantitative Real Time Polymerase Chain Reaction’/exp OR ‘Quantitative Real-Time PCR’/exp OR ‘Protein expression’/exp OR ‘Quantitative assay’/exp OR ‘In Situ Hybridization’/exp OR ‘Hybridization in Situ’/exp

AND

Embryonic model

‘Stem Cell*’/exp OR ‘Progenitor Cell*’/exp OR ‘Mother Cell*’/exp OR ‘Induced Pluripotent Stem Cell*’/exp OR ‘IPS Cell*’/exp OR ‘Human Induced Pluripotent Stem Cell*’/exp OR ‘hiPSC*’/exp OR ‘Fibroblast-Derived Induced Pluripotent Stem Cell*’/exp OR ‘Fibroblast-Derived IPS Cell*’/exp OR ‘Fibroblast Derived IPS Cell*’/exp OR ‘Fetal Stem Cell*’/exp OR ‘Hematopoietic Stem Cell*’/exp OR ‘Hematopoietic Progenitor Cell*’/exp OR ‘Lymphoid Progenitor Cell*’/exp OR ‘Lymphoid Stem Cell*’/exp OR ‘Myeloid Progenitor Cell*’/exp OR ‘Myeloid Stem Cell*’/exp OR ‘Neural Stem Cell*’/exp OR ‘Multipotent Stem Cell*’/exp OR ‘Mesenchymal Stem Cell*’/exp OR ‘Bone Marrow Mesenchymal Stem Cell*’/exp OR ‘Bone Marrow Stromal Cell*’/exp OR ‘Multipotent Bone Marrow Stromal Cell*’/exp OR ‘Adipose-Derived Mesenchymal Stem Cell*’/exp OR ‘Adipose Derived Mesenchymal Stem Cell*’/exp OR ‘Adipose Tissue-Derived Mesenchymal Stem Cell*’/exp OR ‘Adipose Tissue Derived Mesenchymal Stem Cell*’/exp OR ‘Mesenchymal Stromal Cell*’/exp OR ‘Multipotent Mesenchymal Stromal Cell*’/exp OR ‘Mesenchymal Progenitor Cell*’/exp OR ‘Wharton Jelly Cell*’/exp OR ‘Pluripotent Stem Cells*’/exp OR ‘Embryonic Stem Cells*’/exp OR ‘Embryonic Stem Cell*’/exp OR ‘Human Embryonic Stem Cells*’/exp OR ‘hESC*’/exp OR ‘H1-hESC*’/exp OR ‘H9-hESC*’/exp OR ‘Totipotent Stem Cell*’/exp OR ‘Human Umbilical Vein Endothelial Cell*’/exp OR ‘HUVEC Cell*’/exp OR ‘Embryoid Bodies’/exp OR ‘Embryoid Bod*’/exp
