# Supplementary material for: A New Strategy for the Old Challenge of Thalidomide: Systems Biology Prioritization of Potential Immunomodulatory Drug (IMiD)-Targeted Transcription Factors
Source: Int J Mol Sci. 2023 Jul 15;24(14):11515. doi: 10.3390/ijms241411515 (PMC10380514; doi:10.3390/ijms241411515)
Supplement: Supplementary file 1 [file ijms-24-11515-s001.zip › Supplemental Figures and Tables legends.pdf]

Figure S1: Network of the proteins obtained with the literature review, without topological analysis;

Figure S2: Venn Diagram comprising the genes differentially expressed only one time (red) and at least two times (blue), and the IMiD-affected genes, according to the literature review (green);

Table S1: Complete literature review annotations;

Table S2: Network statistics for the proteins obtained with the literature review;

Table S3: Topological analysis for the proteins obtained with the literature review;

Table S4: Details of the transcriptome datasets included in the study;

Table S5: Complete list of genes differentially expressed;

Table S6: Description of gene functions for the genes more times differentially expressed;

Table S7: Gene set enrichment analysis for the differentially expressed genes;

Table S8: Gene ontology overrepresentation analysis for the C2H2 TFs suggested by TRRUST database; Supplemental Material 1: Literature review search terms.
