# Supplementary figures and images for: A New Strategy for the Old Challenge of Thalidomide: Systems Biology Prioritization of Potential Immunomodulatory Drug (IMiD)-Targeted Transcription Factors
Source: Int J Mol Sci. 2023 Jul 15;24(14):11515. doi: 10.3390/ijms241411515 (PMC10380514; doi:10.3390/ijms241411515)

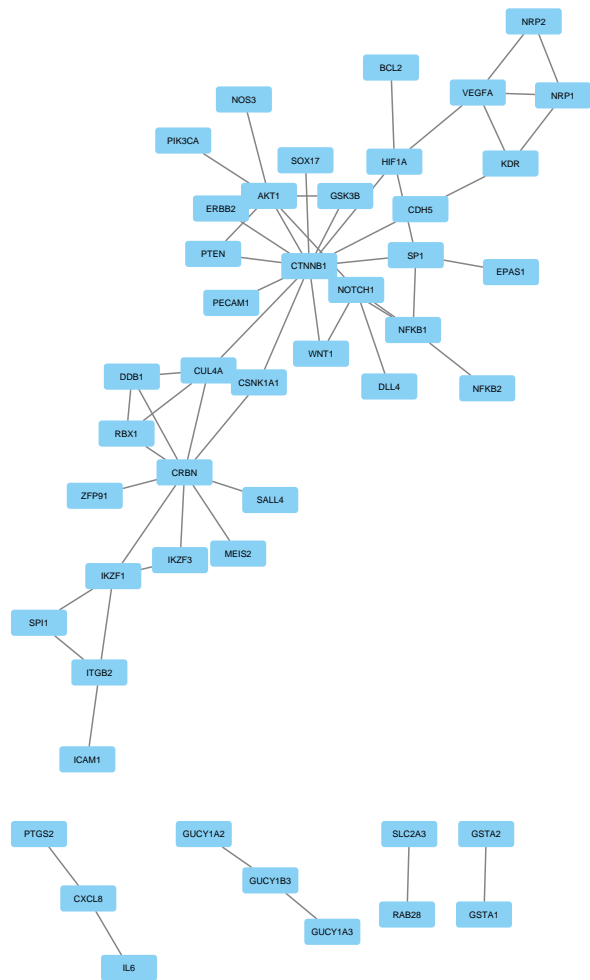

Supplement: Supplementary file 1 [file ijms-24-11515-s001.zip › Kowalski et al., 2023 - Figure S1.pdf]

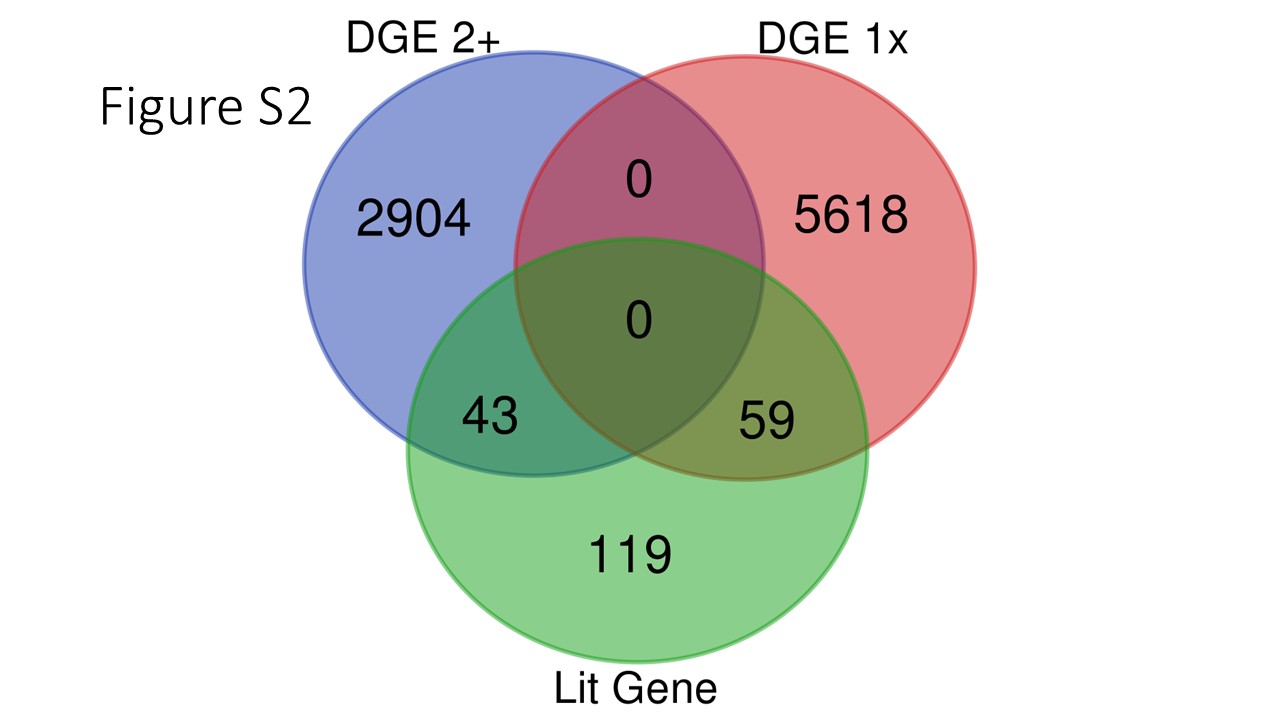

Supplement: Supplementary file 1 [file ijms-24-11515-s001.zip › Kowalski et al., 2023 - Figure S2.jpg]
